# Functional Magnetic Resonance Imaging Analysis of the Clinical Effect and Cerebral Mechanism of Tuina in Lumbar Disc Herniation: Protocol for a Randomized Controlled Parallel Group Trial

**DOI:** 10.2196/63852

**Published:** 2024-09-30

**Authors:** Changzheng Jiang, Hongye Huang, Lechun Chen, Jingjing Jiang, Huanzhen Zhang, Jincheng Chen, Shuijin Chen, Zhigang Lin

**Affiliations:** 1 Fujian University of Traditional Chinese Medicine Fuzhou China; 2 Rehabilitation Hospital Affiliated to Fujian University of Traditional Chinese Medicine Fuzhou China

**Keywords:** fMRI, functional magnetic resonance imaging, lumbar disc herniation, Tuina, traction, transcutaneous electrical nerve stimulation, cerebral mechanism

## Abstract

**Background:**

Lumbar disc herniation (LDH) has become a serious public health and socioeconomic problem. Tuina is a Chinese medicine treatment method based on meridian acupuncture theory and modern anatomy. Tuina can relieve pain and muscle tension and improve functional disorders; this massage is performed by pressing, kneading, pushing, pulling, and shaking the skin, muscles, and bones. However, the mechanism of action and the effect of Tuina as an external treatment on the activities of the central nervous system to relieve LDH pain is unclear. Therefore, we performed functional magnetic resonance imaging (fMRI), which is widely used in pain-related research, as it can detect the effects of different types of pain on brain activity.

**Objective:**

Our randomized controlled parallel-group trial aims to compare the effects of Tuina with those of transcutaneous electrical nerve stimulation (TENS) with traction in patients with LDH.

**Methods:**

This trial will be conducted between May 2024 and April 2025 in the Rehabilitation Hospital affiliated to Fujian University of Traditional Chinese Medicine. Seventy-six participants with LDH will be enrolled for this trial and randomly assigned to 2 groups: Tuina intervention group and TENS with traction intervention group. Participants in both groups will receive treatment for 14 days. fMRI will be performed for the main pain measurements by assessing the effect of the intervention on brain activity before and after the end of the intervention. Short-Form McGill Pain Questionnaire, pressure pain thresholds, and the Oswestry disability index will be used to reflect the degree of pain and lumbar dysfunction, and the results will be used as secondary outcome measurements.

**Results:**

The study protocol has been approved by the ethics review committee of The Rehabilitation Hospital affiliated to Fujian University of Traditional Chinese Medicine. This study was registered on May 1, 2024, with the Chinese Clinical Trial Registry. Data collection began on May 2024 and is expected to end on April 2025. Currently, data from this trial are in the collection phase, and no data analysis has been performed. As of July 1, 2024, we have collected data from 21 patients. The results of this trial are expected to be submitted for publication in September 2025.

**Conclusions:**

This clinical trial will compare the effectiveness of Tuina with that of TENS with traction in the treatment of patients with LDH and will show the cerebral mechanism of Tuina in LDH treatment by using fMRI. The results of our trial will be helpful in clarifying the cerebral mechanism of Tuina in the treatment of LDH and provide a solid foundation for Tuina therapy research.

**Trial Registration:**

Chinese Clinical Trial Registry ChiCTR2400083784; https://www.chictr.org.cn/showproj.html?proj=225157

**International Registered Report Identifier (IRRID):**

DERR1-10.2196/63852

## Introduction

Lumbar disc herniation (LDH) is a common clinical disease, which is caused by the rupture of the annulus fibrosus of the lumbar intervertebral disc, herniation of nucleus pulposus, and stimulation and compression of the dural sac and nerve root, leading to a series of symptoms such as low back pain, lower limb radiation pain, and numbness [1]. LDH is more prevalent among individuals aged 30-50 years, and the lifetime risk for symptomatic LDH is 1%-3%. In contemporary society, due to poor lifestyles and lack of exercise, the incidence of LDH is increasing every year and is frequently reported among younger male populations; therefore, LDH has become a serious public health and socioeconomic problem [2].

The pathogenesis of pain caused by LDH is complex, mainly involving the central and peripheral nervous systems. The peripheral mechanism is usually attributed to mechanical compression or local inflammation of the nerve roots caused by LDH [3]. For example, larger protrusions lead to lumbar spinal nerve root damage and induce changes in nerve fibers, and inflammatory response–related factors result in local inflammation in the microenvironment, leading to LDH pain. At the same time, related inflammatory mediators in LDH, such as interleukin-1β, interleukin-6, interleukin-8, tumor necrosis factor-α, and interferon γ, can also synchronously stimulate peripheral nerve endings and receptors and lead to LDH pain [4,5]. However, the peripheral mechanism alone is not enough to explain the complex clinical symptoms caused by LDH, such as pain sensitization, anxiety, and depression, which are often observed in patients with LDH [6,7]. Therefore, the central mechanism is considered to play a key role in the etiology of LDH.

Functional magnetic resonance imaging (fMRI) is an imaging technique that depicts the neural activity in the brain in real time based on the changes in local oxygen consumption and cerebral blood flow [8]. fMRI is widely used in pain-related research, and it can detect the effects of different types of pain on brain activity. A number of studies have shown that structural and functional changes can occur in the brains of patients with LDH [9]. Among them, a prospective study found that the volume of gray matter volume in the right anterolateral prefrontal cortex, right temporal lobe, left premotor cortex, right caudate nucleus, and the right cerebellum decreased significantly, while the volume of the right dorsal anterior cingulate cortex, left precuneal cortex, left fusiform gyrus, and the right brainstem increased in patients with LDH, suggesting that LDH can cause specific local changes in the brain structure [10]. In patients with LDH, the amplitude of low-frequency fluctuations (ALFF) increased in the right lingual gyri in the conventional band and in the left Cerebelum_Crus1 in the slow-4 band [11]. In addition, patients with LDH also have impaired excitability of the somatosensory and motor cortex, decreased homogeneity of the default mode network, and dysfunction of functional connectivity (FC) [12]. These findings suggest that brain structural remodeling and functional changes may be the cerebral mechanism of the pathogenesis of LDH.

At present, the treatment of LDH mainly includes conservative treatment, interventional therapy, and surgical treatment [13]. Tuina is a massage therapy guided by traditional Chinese medicine theory, which has a long history. Recently, several randomized controlled trials have confirmed the positive effects of Tuina treatment [14-16]. For example, Tuina has a good therapeutic effect on diseases such as LDH, chronic neck pain, and knee osteoarthritis. A meta-analysis of 13,075 patients also showed that Tuina is more effective than traction and Chinese herbal medicine in the treatment of LDH [17]. However, the mechanism of Tuina in the treatment of pain in LDH has not been fully elucidated, and the potential cerebral mechanism of Tuina analgesia is a hot topic of interest.

Recently, some scholars have used fMRI technology to study the central mechanism of Tuina in the treatment of LDH pain and obtained some positive results. For example, Zhou et al [18] found that patients with LDH showed abnormal brain ALFF and regional homogeneity (ReHo) values, which were altered after Tuina manipulation. Patients with LDH showed decreased ReHo in the left orbital part of the middle frontal gyrus, decreased dynamic FC (dFC) variance between the left orbital part of the middle frontal gyrus and the left fusiform, and increased dFC variance in the left orbital inferior frontal gyrus and left precuneus. Tuina therapy positively altered the ReHo and dFC values, making the brain activities in patients with LDH similar to those in healthy controls [12].

In recent years, although many studies [12,19] have provided evidence on the central brain mechanism of Tuina analgesia by using fMRI technology, relatively few studies have explored the central mechanism of Tuina in the treatment of LDH. Therefore, we designed a randomized controlled trial to explore the potential cerebral mechanism of Tuina analgesia by using fMRI.

## Methods

### Trial Design

**Figure 1:**
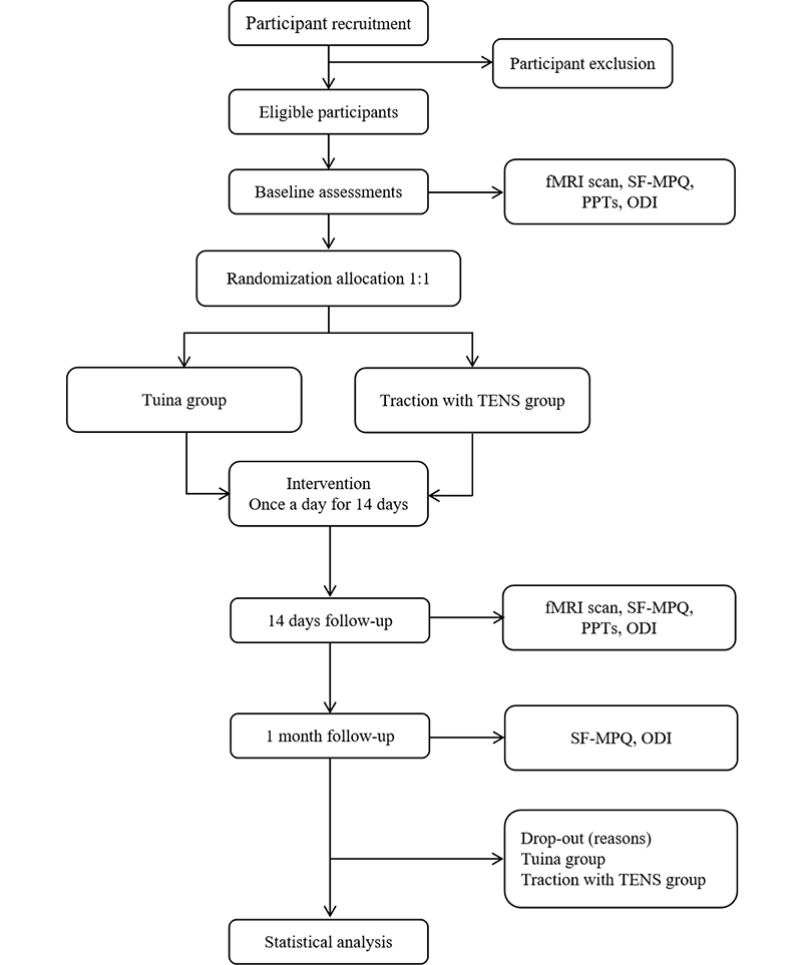
Flowchart of this trial. fMRI: functional magnetic resonance imaging; ODI: Oswestry disability index; PPT: pressure pain threshold; SF-MPQ: Short-Form McGill Pain Questionnaire; TENS: transcutaneous electrical nerve stimulation.

**Figure 2:**
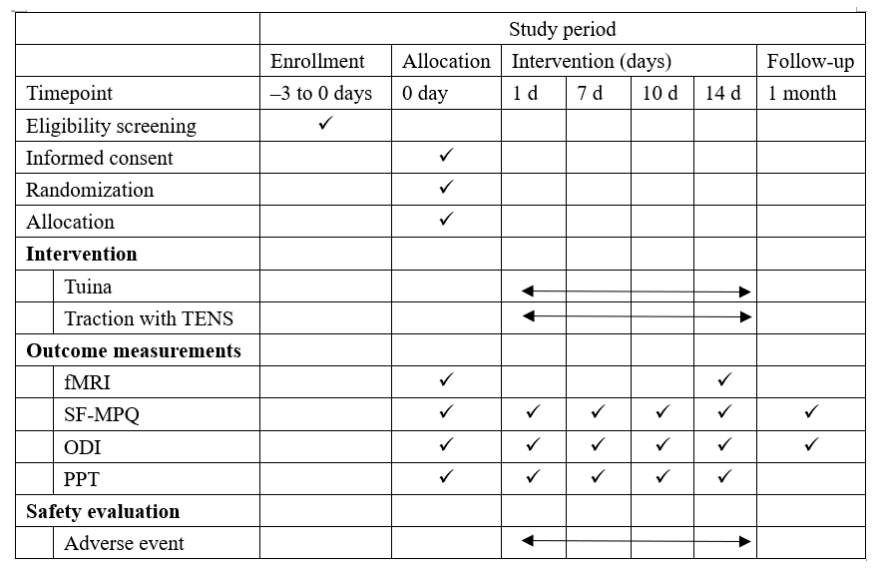
Flow diagram of this study. –3 to 0 days: within 3 days prior to treatment; 0 day: day 0 of treatment; 1 day: day 1 of treatment; 7 days: day 7 of treatment; 10 days: day 10 of treatment; 14 days: day 14 of treatment; 1 month: 1 month after treatment; fMRI: functional magnetic resonance imaging; ODI: Oswestry disability index; PPT: pressure pain threshold; SF-MPQ: Short-Form McGill Pain Questionnaire; TENS: transcutaneous electrical nerve stimulation.

This is a single-center randomized controlled trial with 2 parallel arms: the Tuina intervention group and traction with transcutaneous electrical nerve stimulation (TENS) intervention group. A total of 76 eligible patients with LDH will be recruited for this trial, and patients with LDH will be randomly assigned to the groups in 1:1 ratio. All patients will provide written informed consent. Outcome evaluation and statistical analysis will be performed by 2 researchers who do not know the specific groups. The trial design flowchart is shown in Figure 1, and the study schedule is shown in Figure 2. This study will be conducted in the Rehabilitation Hospital affiliated to Fujian University of Traditional Chinese Medicine. This study follows the CONSORT (Consolidated Standards of Reporting Trials) guidelines (Multimedia Appendix 1).

### Ethics Approval

The study protocol has been approved by the ethics review committee of The Rehabilitation Hospital affiliated to Fujian University of Traditional Chinese Medicine (project 2024KY-008-02). This study was registered on May 1, 2024, with the Chinese Clinical Trial Registry (ChiCTR2400083784).

### Participants and Recruitment

We will recruit participants in the Rehabilitation Hospital affiliated to Fujian University of Traditional Chinese Medicine. All participants need to meet the LDH diagnostic criteria in the guidelines for clinical diagnosis and treatment (Orthopedic Bundle Separation) [20]. In addition, we will recruit potential patients through posters and internet platforms. Clinical trial communicators will fully explain the trial to patients before the trial begins to ensure that all the patients participate voluntarily and sign an informed consent form.

### Inclusion Criteria

The inclusion criteria for the participants are as follows: (1) right-handed; (2) age between 18 and 60 years; (3) computed tomography or magnetic resonance imaging examination show L4-L5 or L5-S1 disc herniation; (4) the main manifestation is nerve root pain, and the imaging is consistent with the nerve localization; (5) the neuropathic pain rating scale (Douleur Neuropathique 4 questions) score is ≥4; (6) patients who did not take painkillers or nutritional neurodrugs for nearly 1 month and did not receive systematic treatment; and (7) patients who voluntarily want to participate in clinical trials and sign informed consent forms.

### Exclusion Criteria

The exclusion criteria are as follows: (1) patients with progressive neurological deficit or cauda equina syndrome; (2) patients with LDH combined with other major lumbar diseases such as tumor, tuberculosis, and severe osteoporosis; (3) patients with trauma, acute lumbar injury caused by surgery pain, and lower limb nerve injury; (4) patients with any other condition causing lower limb neuropathy (diabetes or neurological diseases); (5) patients with cardiovascular, pulmonary, renal, hematopoietic system, and other major diseases; (6) patients with mental health issues; (7) pregnant or lactating women; and (8) patients with contraindications for fMRI.

### Dropout Criteria

Participants who were unable to complete the trial for the following reasons will be considered to have dropped out: (1) patients are not treated by the regulations, and the curative effect cannot be judged; (2) incomplete data that affect the judgment of efficacy or safety; or (3) accidents occur in the course of treatment and participants are unable to adhere to treatment.

### Comprehensive Suspension Criteria

The comprehensive suspension criteria are as follows: (1) participants in the study have serious adverse effects due to a poor treatment plan, (2) participants experience severe complications or disease deterioration during the study period, and (3) if the test termination conditions are met after the doctor follows the standard operating procedure treatment, they shall be terminated immediately.

### Randomization

An independent technician will use the SPSS statistical software (version 26.0; IBM Corp) to generate random sequence numbers, and participants will be randomly divided into the Tuina intervention group or the traction with TENS intervention group in 1:1 ratio. Random sequence numbers will be sealed in opaque envelopes. The envelopes will be opened in a numerical order to determine the allocation for the participants.

### Blinding

In this study, blindness will be implemented for outcome evaluators and data statisticians. Due to the particularity of the treatment method in this trial, the therapists and patients will not be blinded for their grouping. This blinded process will continue until the end of the trial data analysis.

### Interventions

Participants will receive treatment once a day for 14 days. Tuina treatment will be performed by a professionally trained Tuina therapist, and a senior attending physician will perform TENS and traction. Before the trial, both operators should pass the relevant clinical tests to ensure the operation’s consistency. Other related treatments such as medicines, acupuncture, and physiotherapy will not be allowed during the treatment period.

### Tuina Intervention Group

**Figure 3:**
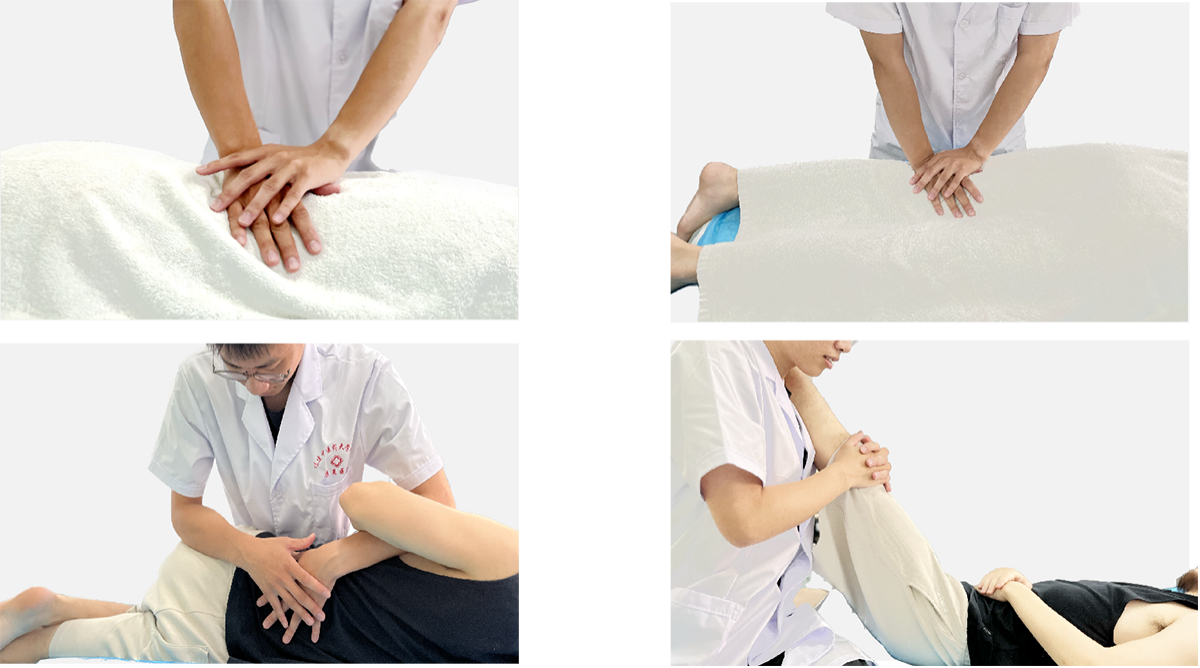
Operation in the Tuina intervention group.

The standard Tuina procedure will be based on Tuina therapy described in [21]. The specific therapy will be performed as follows (Figure 3).

1. The Tuina therapist will guide the patient to lie in the prone position and gently press the bladder meridian on both sides of the patient’s waist and buttocks as well as the posterior lateral side of the affected lower limb for 10 minutes of operation.
2. Thereafter, the Tuina therapist will rhythmically press the acupoints of Shenshu (BL23), Dachangshu (BL25), Huantiao (GB30), Chengfu (BL36), Weizhong (BL40), Chengshan (BL57), and Kunlun (BL60) with their fingers at a frequency of 120 times per minute for 10 minutes of operation.
3. Then, the Tuina therapist will guide the patient to lie on their side by using the waist oblique pull method once on the left and once on the right.
4. Finally, the patient is placed in a supine position, and the lower limb on the affected side is raised. The operation is similar to a straight leg lift test, and this process will be repeated several times.

### TENS With Traction Intervention Group

#### Step 1: TENS

The patient lies in the prone position on the treatment bed. In order to treat the patients, the therapist places 2 electrodes of the TENS therapeutic apparatus (WOND2000F0, SUNJAVA) on the Ashi acupoint on the patient’s back and lower limbs. The therapist then changes the stimulation parameters, setting the frequency to 25 Hz and the pulse width to 280 µs. The therapist chooses the maximum intensity based on the patient’s condition and the level that the patient can tolerate. TENS is performed once a day for 20 minutes, and the treatment will be continued for 14 days.

#### Step 2: Traction

Traction will be performed after 10 minutes of TENS. The patient lies on the spine traction bed (TC-30S, MINATO). Once the therapist assists the patient in putting on the traction strap, they determine the traction amount based on the patient’s weight. Initially, the therapist sets the traction volume at 25% of the patient’s body weight, gradually increasing it by 2 kg/time as the patient’s condition changes until it reaches 40%-50% of the patient’s body weight. The therapist allows the patient to lie down and rest for 5 minutes after each traction session. The patient will receive traction once a day, with a duration of 20 minutes each time. The treatment will last for 14 days.

### Outcome Measurements

We will present the trial results by using both objective and subjective indicators. fMRI will be used for the main measurements, reflecting the changes in the participants’ brain structure and functional changes. Short-Form McGill Pain Questionnaire (SF-MPQ), pressure pain thresholds (PPTs), and the Oswestry disability index (ODI) will indicate the degree of pain and lumbar dysfunction and will be used for secondary outcome measurements.

### Primary Outcome Measurement

The fMRI examination will be performed in the Imaging Department of the Rehabilitation Hospital affiliated to Fujian University of Traditional Chinese Medicine. The first fMRI scan will be performed 1 day before the start of treatment, and the second fMRI scan will be performed within 6 hours after the last intervention. The fMRI data will be collected by a 3.0 Tesla magnetic resonance scanner (MAGNETOM Prisma 3.0T, Siemens) with 64-channel phase-array head coils. During the acquisition process, participants will be required to close their eyes, relax, stay awake, keep their heads fixed as far as possible, and not perform thinking activities. We will perform fMRI at the beginning and end of the experiment.

To acquire the structural images, we will use the following parameters. The T1-weighted imaging scan parameters are as follows: time of repeat=2200 ms, time of echo=2.48 ms, flip angle=8°, field of view=230 mm×230 mm, matrix=256×256, resolution=0.98×0.98×1 mm^3^, slice number=160, and slice thickness=1.0 mm. The functional images will be acquired using the following parameters: time of repeat=2000 ms, time of echo=30 ms, flip angle=90°, field of view=230 mm, matrix=64×64, resolution=3.6×3.6×3.6 mm^3^, slice number=37, slice thickness=3.6 mm, and scan time=10 minutes for 300 timepoints.

### Secondary Outcome Measurements

#### SF-MPQ Indicators

We will translate the SF-MPQ into Chinese to assess pain perception. This questionnaire consists of 5 indicators [22]: sensory pain rating index, affective pain rating index, total pain rating index, visual analog scale (which assesses the overall intensity of the total pain experience), and present pain index. The sensory pain rating index consists of 11 items describing sensory pain, and the affective pain rating index consists of 4 items describing emotional pain, with scores of 0 (none), 1 (mild), 2 (moderate), and 3 (severe). The total pain rating index is the sum of the sensory pain rating index and affective pain rating index. The overall intensity assessment of total pain experience consists of a visual analog scale ranging from 0 to 10, with 0 indicating no pain and 10 indicating the most severe pain imaginable [23]. In addition, 6 levels appear in the present pain index to describe the patient’s pain level: no pain, mild, uncomfortable, painful, terrible, and unbearable expressed as 0, 1, 2, 3, 4, and 5 points, respectively, for each participant. We will ask participants to indicate the score that best matches their level of pain.

#### PPT Test

**Figure 4:**
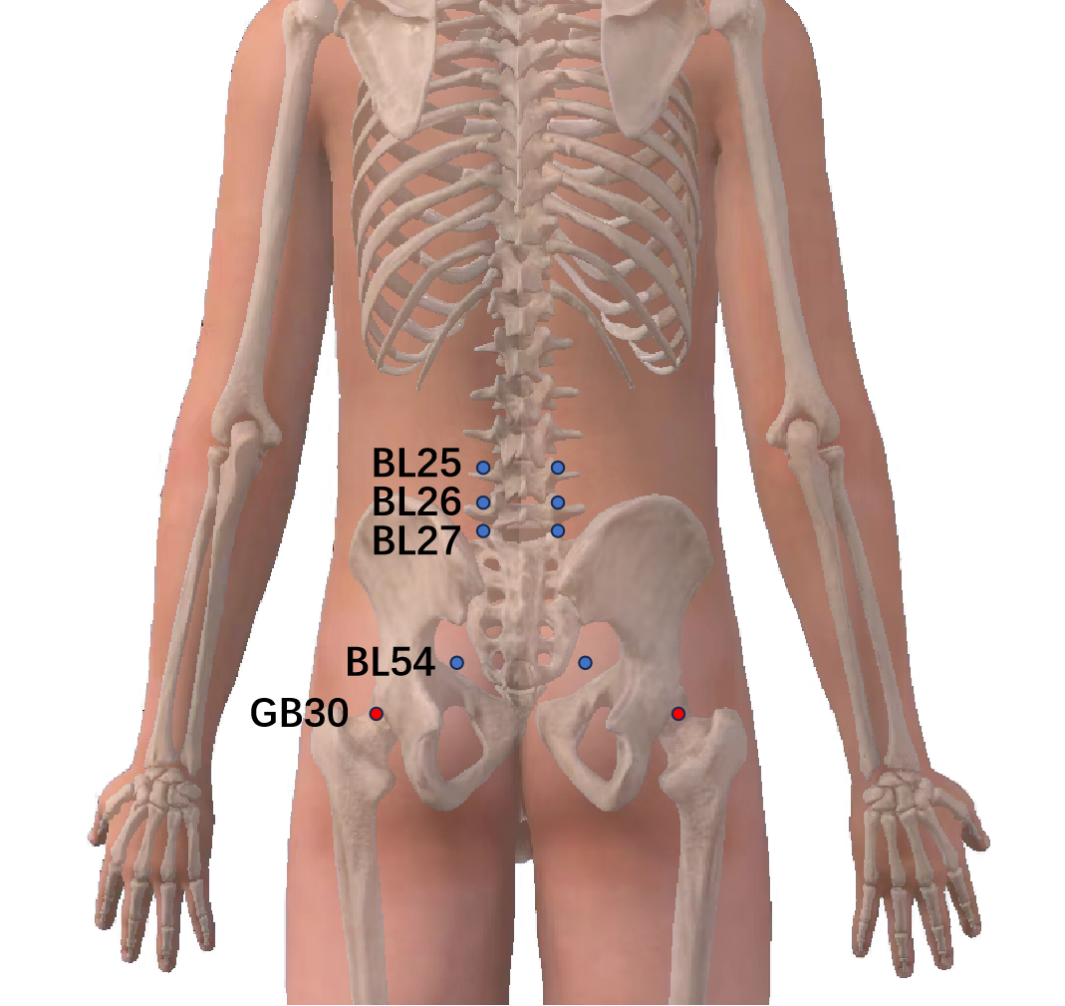
Test points used to measure the pressure pain threshold. BL25: Dachangshu; BL26: Guanyuanshu; BL27: Xiaochangshu; BL54: Zhibian; GB30: Huantiao.

PPT is a quantitative sensory test that can effectively describe the intensity of pain [24]. When the patient lies in the prone position, the estimator first locates the Dachangshu (BL25), Guanyuanshu (BL26), and Xiaochangshu (BL27) on the patient’s erector spinae muscles and Zhibian (BL54) and Huantiao (GB30) on the gluteus maximus muscles and uses them as test points (Figure 4). Then, the estimator will use the handheld tenderness tester (FTX50, Wagner) to press the test points at a uniform speed, lock it immediately when the patient feels pain, and then record the pressure value. The PPT will be measured every 5 minutes; a total of 3 measurements will be recorded, and the average will be taken to indicate the PPTs.

#### ODI Questionnaire

The ODI is a commonly used questionnaire for self-quantification of dysfunction in patients with LDH, and it has good reliability and sensitivity in patients with LDH [25]. This questionnaire consists of 10 questions. The score for each question ranges from 0 to 5. The maximum total score is 45 points. ODI score = [actual score/(5×number of actual answered questions)]×100%. The higher the score, the more severe the dysfunction.

#### Safety Evaluation

The investigators will assess the adverse events, defined as unexpected or adverse reactions occurring during or after treatment. In this experiment, adverse events [26] will be defined as (1) syncope, (2) ecchymosis, (3) increased pain, (4) fracture, and (5) skin allergy. The investigator will assess and solve the adverse events during the study, reporting them to the appropriate departments and ethics committees. Following that, they will monitor all the adverse events until the event resolves or the participant’s condition becomes chronic or stable, and they will make every effort to ensure their safety and record the relevant information in case report forms.

#### Follow-Up

To evaluate the efficacy and safety of the intervention, we will follow up with the participants for 1 month after the end of the treatment. A month after the treatment ends, the results evaluator will call the participants to investigate the recurrence of their symptoms. Participants can also inform evaluators of their clinical symptoms face-to-face or via email, text message, or WeChat at the relevant timepoint.

#### Data Collection and Monitoring

The outcome assessors will record the detailed information in case report forms, including the evaluation of treatment effectiveness, adverse events, questionnaire data, and fMRI data. Two data administrators outside the team will use the Excel database for data entry and management, and these data administrators must complete rigorous training in data monitoring. To ensure the accuracy of the data, 2 data administrators will enter the information independently and proofread it, and they will enter the real-time data into the China Clinical Trial Registration Center. During the trial, the Rehabilitation Hospital affiliated to Fujian University of Traditional Chinese Medicine will review the conduct of the trial.

### fMRI Data Preprocessing

First, MRIConvert 2.0 software (Lewis Center for Neuroimaging) converts the DICOM format of the fMRI images into a Neuroimaging Informatics Technology Initiative format file for data analysis. Next, we apply the default preprocessing pipeline in the Statistical Parametric Mapping–based CONN 2.0b toolbox of the MATLAB 2022b (The MathWorks, Inc) platform to preprocess all the nuclear magnetic data.

### fMRI Data Processing

#### ALFF Analysis

The ALFF analysis will be performed using the AFNI (Analysis of Functional NeuroImages) software [27]. The fast Fourier transform transforms the filtered time series to the frequency domain (parameters: taper=0%, fast Fourier transform length=shortest) to obtain the power spectrum. Since the power of a given frequency is proportional to the square of the magnitude of this frequency component in the original time series, the square root is calculated at each frequency of the power spectrum, and the average square root ranges from 0.01 Hz to 0.08 Hz at each voxel. This average square root is considered as the ALFF.

#### ReHo Analysis

The Kendall coefficient of concordance is used to measure the local synchronization of the time series of its nearest neighbor voxels as 
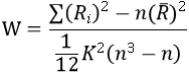
 [28], where W is the Kendall coefficient of concordance among given voxels, ranging from 0 to 1; Ri is the sum rank of the ith timepoint; R (n+1) K/2 is the mean of Ri; K is the number of time series within a measured cluster (K=7, 19, and 27; K=27 is used in this study); and n is the number of ranks (here n=60). The Kendall coefficient of concordance program is coded in MATLAB [29]. Thus, an individual ReHo map is generated for each data set.

#### FC Analysis

Differential brain regions obtained by ALFF or ReHo analysis, regions of interest, and differential brain regions will be selected, and FC will be calculated. An FC plot for each participant will be obtained by DynamicBC using the sliding window method. We will select a window length of 50 times of repeat (time of repeat: 100 seconds) with a window overlap of 98% (step size of 1 time of repeat) to calculate the FC for each participant. The FC plots for each participant will be calculated within each window to generate a series of FC plots. The coefficient of variation of the FC map is calculated to measure the temporal variability of intrinsic brain activity. Finally, the FC variability of all the participants will be transformed into standardized *z* scores to enhance the normality of the data by subtracting the mean of each voxel and dividing by the standard deviation [30].

### Statistical Analysis

Statistical analysis will be conducted using SPSS 26.0 statistical software. Count data will involve the ratio of the sex and age variables compared between the 2 groups via the *χ*^2^ test of the 4-fold table or the Fisher test. The SF-MPQ scores, PPTs, and ODI scores are the rank data. Therefore, the Mann-Whitney *U* test will be used for the intergroup comparison, and the Wilcoxon symbolic test will be applied for the intragroup comparison. Paired 2-sided *t* test will be used to assess the difference in ALFF and ReHo between the 2 groups. In addition, Pearson correlation coefficients will be used to estimate the connection between the seed region and all of the voxels within the whole brain separately based on the patients. Statistical significance will be set at *P*<.05 (2-sided).

### Sample Size Calculation

There is still no consensus on sample size estimates for fMRI studies. Desmond and Glover’s [31] empirical research results indicate that to achieve 80% test power at the single voxel level in magnetic resonance imaging data with a multiple comparison correction threshold of 0.05, at least 24 participants are required. We also referred to similar studies investigating neuroimaging mechanisms related to LDH and combined them with the research design for this topic [12,18]. The visual analog scale was selected as an important indicator of LDH pain level [32]. According to literature [33], patients with LDH had a score of 4.28 (SD 1.16) points after Tuina intervention and a score of 5.68 (SD 1.26) points after traction intervention. We used G*Power 3.1 software to calculate the sample size based on the mean, wherein α is .05 and power is .85. Each of the 2 groups require 30 cases. With 20% dropout, 38 cases are required for each group for a total of 72 cases.

### Quality Control

#### Quality Control of the Evaluation Methods

Before the implementation of this study, we conducted centralized and unified training for all participants to achieve consistency in their understanding and mastery of the criteria (diagnosis, inclusion, exclusion, elimination, termination criteria, etc), the operating procedures of intervention measures, observation index collection and scoring criteria; formulation of a detailed research plan and schedule; assessment indicators; and strict and consistent implementation of the study plan.

#### Quality Control of the Research Participants

Before the study begins, we will provide each participant with details about the operability and precautions of the intervention method, prohibit other treatment methods during the intervention, and provide free health counseling throughout the study. With the informed consent of the participants, the corresponding test contents can be included and conducted voluntarily. If the pain fails to be relieved during the intervention, the project team must be informed in time and relevant drugs must be given if necessary; if the symptoms cannot be relieved, comprehensive treatment should be given immediately. If there is an accident or adverse event during a treatment event, the record form should be filled accurately, providing details such as the adverse event type, occurrence time, severity, duration, measures taken, and other reports.

#### Quality Control of Data Management

The case observation form should be as complete and accurate as required, avoiding random alteration. The same person should conduct the observation records of the same participant, ensure consistency between the recorded data in the computer database and the original data, and correctly select the statistical analysis method.

#### Quality Control of Tuina

Tuina will be performed by a physician with more than 2 years of clinical experience in Tuina therapy, who has received specialized Tuina therapy training and passed relevant tests. The physician is required to perform specific Tuina operations according to the operating instructions and maintain consistency in the Tuina operations.

## Results

This trial was approved by the ethics committee of the Rehabilitation Hospital affiliated to Fujian University of Traditional Chinese Medicine (project 2024KY-008-02). This trial is in the participant recruitment and intervention phase. Participant enrollment began in May 2024, and data collection is expected to end on April 30, 2025. As of July 1, 2024, we have collected data from 21 cases. The results of this trial are expected to be submitted for publication in September 2025.

## Discussion

### Overview

This randomized controlled trial investigates the clinical efficacy and potential mechanism of Tuina in treating LDH. The results of this trial will be helpful in clarifying the cerebral mechanism of Tuina in the treatment of LDH. LDH occurs when the annulus fibrosus of the lumbar intervertebral disc tears, the nucleus pulposus herniates, and the dural sac and nerve root are stimulated and compressed [1]. This causes a wide range of symptoms, including low back pain, radiation pain in the lower limbs, and numbness. Pain, the primary symptom of LDH, is usually the main reason for patients to seek medical treatment [34].

Tuina is a traditional Chinese medicine treatment method based on meridian acupoint theory and modern anatomy [35]. A therapist manipulates the patient’s skin, muscles, and bones with both hands, including pressing, kneading, pushing, pulling, and shaking. Tuina is widely used for the treatment of LDH in China because of its proved effectiveness in relieving pain and muscle tension and improving dysfunction [35,36]. Traction is a technique used for stretching soft tissue and separating joint surfaces or bones to achieve the treatment goal [37]. Through electrodes placed on the skin, TENS creates pulsed alternating current. This can help relieve pain by activating the central nervous system’s endogenous inhibition mechanism [38-40]. The *Chinese Journal of Evidence-Based Medicine* published the “Evidence-based practice guide for nonoperative treatment of LDH” in 2024, listing Tuina, traction, and TENS as routine treatments and interventions for LDH [41].

Pain is considered to be an unpleasant and emotional feeling related to actual or potential histopathology [42]; therefore, changes in pain perception should include subjective and objective indicators. This study will use subjective SF-MPQ and ODI scores to assess changes in pain perception and dysfunction, while the objective index of tenderness threshold will demonstrate changes in pain symptoms. The combination of subjective and objective indicators can comprehensively reflect the changes in the symptoms of patients with LDH, reducing the occurrence of potential bias and improving the quality of clinical evidence.

In addition, we add neuroimaging observations based on objective indicators. Previous neuroimaging studies have shown that patients with LDH can show changes in brain structure and function. Liu et al [43] found that compared with the control group, patients with LDH had considerably longer typical path lengths in the brain network, as well as worse clustering coefficients, global efficiency, and local efficiency. Another systematic review [44] noted that in patients with LDH, the medial prefrontal cortex, cingulate cortex, amygdala, and insular lobe are more active. Pain-relief areas are less active, and the FC in pain-related areas is changed. Mei et al [45] reported that LDH may involve altered FC in several brain regions as well as decreased excitability in sensory motor areas during tasks and increased activity in the sensory-motor network during resting states. Therefore, brain structural changes and dysfunction caused by long-term pain afferent signals may be the key factors for pain associated with LDH.

Neuroimaging technology also provides an objective basis for Tuina’s cerebral mechanism in treating LDH. Li et al [46] found that after receiving Tuina treatment, the hypothalamus, left nucleus accumbens, and left amygdaloid of patients with LDH were excited, while the left anterior cingulated gyrus was inhibited. Yuan et al [47] discovered that after Tuina treatment, the brain functional activity in patients with LDH was mainly inhibited and the inhibited areas were located in the right side of the prefrontal cortex and cerebellum and that Tuina has the ability to alter the default mode network’s functionality in patients with LDH, which may help explain some of the intervention’s analgesic effects [12]. The above findings suggest that brain function regulation may be an important factor affecting Tuina’s effect on pain regulation. Therefore, we designed this study as a clinical randomized controlled trial to compare the effectiveness of Tuina with that of traction combined with TENS in patients with LDH and to investigate the cerebral mechanism of Tuina in LDH treatment by using fMRI. The results of this trial will provide a visual basis for the clinical application of Tuina in the treatment of LDH and provide a solid foundation for Tuina therapy research.

### Study Limitations

In the course of operation, due to the differences in the form of treatment between Tuina and traction combined with TENS, it is impossible for participants and operators to be blinded. For this limitation, we will perform quality control on the blindness of the questionnaire and magnetic resonance imaging data analysis to improve the credibility of the questionnaire measurement, which is composed of outcome assessors, data administrators, and data analysts.

### Conclusions

This trial aims to investigate the cerebral mechanism of Tuina in the treatment of LDH. We used TENS combined with traction as a control intervention. We expect that Tuina can relieve pain symptoms in patients with LDH and that this pain relief mechanism is related to the activation or inhibition of pain-related regions in the brain.

## Appendix

Multimedia Appendix 1 CONSORT (Consolidated Standards of Reporting Trials) checklist.

## Abbreviations

AFNI: Analysis of Functional NeuroImages
ALFF: amplitude of low-frequency fluctuations
CONSORT: Consolidated Standards of Reporting Trials
dFC: dynamic functional connectivity
FC: functional connectivity
fMRI: functional magnetic resonance imaging
LDH: lumbar disc herniation
ODI: Oswestry disability index
PPT: pressure pain threshold
ReHo: regional homogeneity
SF-MPQ: Short-Form McGill Pain Questionnaire
TENS: transcutaneous electrical nerve stimulation

## Data Availability

The data sets used and analyzed during this study are available from the corresponding author upon reasonable request.
